# Association between body image dissatisfaction and body anthropometric indices among Chinese children and adolescents at different developmental stages

**DOI:** 10.3389/fpubh.2022.926079

**Published:** 2022-12-13

**Authors:** Yuanyuan Wang, Ruiyao Cao, Xingwang Peng, Li Zhang, Zizhe Zhang, Lianguo Fu

**Affiliations:** Department of Children and Adolescent Health, School of Public Health, Bengbu Medical College, Bengbu, China

**Keywords:** body image dissatisfaction, body shape, body anthropometric indices, puberty, developmental stages

## Abstract

**Objective:**

Children at different developmental stages show different physical development and psychological cognitive characteristics and may pay different attention to body parts. The purpose of this study was to analyze the associations between body image dissatisfaction (BID) and body anthropometric indices (BAIs) among Chinese children and adolescents at different developmental stages.

**Methods:**

A total of 609 Chinese primary and secondary school students aged 8–15 years (329 boys and 280 girls) were selected using stratified cluster sampling. The students' body height, sitting height (SH), weight, chest circumference (CC), hip circumference (HC), waist circumference (WC), scapular skinfold thickness (SST), triceps skinfold thickness (TST), and abdominal skinfold thickness (AST) were measured. Boys' testicular volumes and first spermatorrhea and girls' breast measures and menarche were assessed using the Tanner stage standard. A body shape questionnaire (BSQ) was used to survey the subject's BID.

**Results:**

In boys with testicular volume < 4 ml, the hip-to-height ratio (HHR) was positively correlated with BSQ score (β = 8.17, *P* < 0.01). In boys with testicular volume ≥4 ml and nonfirst spermatorrhea, the HHR and SST were positively correlated with BSQ score (β = 2.51, *P* = 0.04; β = 4.98, *P* < 0.01). In boys with first spermatorrhea, weight was positively correlated with BSQ score (β = 10.30, *P* < 0.01). In girls with breast development < Tanner stage II, waist-to-height ratio (WHtR) was positively correlated with BSQ score (β = 5.12, *P* < 0.01); In girls with breast development ≥ Tanner stage II and nonmenarche, chest-to-sitting height ratio (CSHR) was positively correlated with BSQ score (β = 10.82, *P* < 0.01), and waist-to-hip ratio (WHR) was negatively correlated with BSQ score (β = −3.61, *P* = 0.04). In girls with menarche, WHtR and sitting height-to-height ratio (SHHR) were positively correlated with BSQ score (β = 6.09, *P* < 0.01; β = 2.05, *P* = 0.02).

**Conclusion:**

The associations between body image dissatisfaction and anthropometric indices among Chinese children and adolescents at different developmental stages are different.

## Introduction

Body image is a mental image formed by the size and shape of the body (1). BID is defined as a negative subjective evaluation of one's own body (2), and it occurs when a person has persistent negative thoughts and feelings about their body, which is an internal emotional and cognitive process. Body image dissatisfaction scales are usually used to assess BID and include multidimensional body-self association questionnaires, body esteem scales, satisfaction and dissatisfaction with body parts scales, and body shape questionnaire (BSQ) (3). Previous studies have shown that BID is associated with a range of adverse health outcomes, including obesity (4), low self-esteem (5), depression (6), eating disorders (7), and quality of life aspects related to psychosocial functioning and self-perception concepts (2). The incidence of BID has been reported as 35% to 81% in girls and 16% to 55% in boys (8). Since BID has become an important public health problem affecting the physical and mental health of children and adolescents, an increasing number of people have been aware of the universality of children's dissatisfaction with body image (9).

Children's body anthropometric indices (BAIs) are the basis of dissatisfaction with their own body (10). Under the influence of nearby companions (11), parents (12), and media (13), children and adolescents have dissatisfaction with their body more easily. Several studies have begun to pay attention to the relationships between obesity and BID (14). The rate of BID among overweight or obese girls is 2–3 times higher than that among normal-weight girls (15–18). However, studies have shown that children and adolescents are dissatisfied with some parts of their bodies, such as waist circumference, leg length, and hips (15). There have been few studies on the relationships between BAIs and BID, especially the indicators reflecting the longitudinal dimension of the body and the indicators reflecting the sexual characteristics of adolescent development, such as chest circumference and testicular volumes (19).

In addition, the physical development of children and adolescents is not only a continuous process but also a phased feature. Children with different developmental stages not only have different developmental characteristics but also may pay different attention to the body. A study showed that there were differences in body-shape distortion between different ages (20). Zhang et al. (21) reported that girls were more dissatisfied with their sexual organs before breast development reaches Tanner stage II and more dissatisfied with their gender after menarche. The study showed that there was a view of “thin for beauty” in early childhood, even during childhood (22). The girls who have started puberty may pay increasing attention to their breast development, waist circumference, legs and hips (23, 24), while boys who have started puberty may expect more muscle gain (19). Girls with menarche or boys with first spermatorrhea are more concerned with their body image as they begin to pay attention to the perceptions of their parents and the opposite sex, which are influenced by culture, media, and idols (21).

As seen above, body anthropometric indices related to body image cognition may be different at different pubertal developmental stages. The purpose of this study was to analyze the associations between body image dissatisfaction and anthropometric indices among Chinese children and adolescents at different developmental stages. It is important to guide children and adolescents to identify and accept their bodies correctly.

## Materials and methods

### Participants

In this study, 609 students (329 boys and 280 girls) aged 8–15 years were selected from primary and secondary schools in Bengbu using stratified cluster sampling (stratified by grade and grouped by class). The research protocols for this study were approved by the Bengbu Medical College Medical Research Ethics Committee (2015 No. 003), and all subjects' guardians signed informed consent before the examination and questionnaire survey were performed.

### Anthropometrical measurements, indices, and detection of indicators of puberty development

The medical staff measured participants' anthropometric indices including body height, SH, weight, CC, HC, WC, SST, TST, and AST. A mechanical height meter (HLZ-51 height sitting height meter, Huatou (Tianjing) electronic technology Co., Ltd, 0.1 cm) was used to measure the subjects' height and SH. When measuring height and SH, the subjects took off their shoes and hats, kept upright, and looked forward. An electronic weight meter (XMTZC05HM xiaomi weight scale, Xiaomi Co., Ltd, 0.1 kg) was used to measure the subjects' weight. When measuring weight, subjects wore short underwear (girls could wear a thin bra or vest). A tape measure (Gnjz-4067 tape measure, Beijing hystic new materials Co., Ltd, 0.1 cm) was used to measure subjects' CC, WC, and HC. CC is the horizontal circumference along the level of the nipple and subscapular angle. HC is the longest circumference of the hip. WC is the circumference of 1 cm above the navel horizontal line. A skinfold thickness meter (JK6113 skinfould thickness meter, Beijing jingkaida instrument Co., Ltd, 0.5 mm) was used to measure the subject's SST, TST, and AST. The inferior angle of the scapula is where the vertebral (medial) and axillary (lateral) borders intersect, and SST is a diagonal fold just below the inferior angle of the scapula. TST is a vertical fold midway between the acromion process and the olecranon process (elbow). The AST is the thickness of the skin fold at the intersection of the right clavicular midline and the horizontal line.

### Detection of the measurements related to pubertal stages

The testicular volume of boys was measured using a testicular volume meter and the boys were asked whether the first spermatorrhea had occurred. The breast development of girls was measured based on the Tanner stage standard and were asked whether menarche had occurred. According to the Tanner stage standard, after secondary sex characteristic development and first spermatorrhea or menarche, the pubertal development stages are divided into three stages, including stage 1 (girls: breast development < Tanner stage II; boys: testicular volume <4 ml); stage 2 (girls: breast development ≥ Tanner stage II and nonmenarche; boys: testicular volume ≥ 4 ml and nonfirst spermatorrhea); and stage 3 (girls: after menarche; boys: after the first spermatorrhea).

### Body image dissatisfaction (BID)

A body shape questionnaire (BSQ) was used in our study to survey BID (25). The scale, known as BSQ-14, has 14 items and is a shortened version of the original 34-item BSQ. It is highly recommended for use in assessing BID in field applications and research, and has high reliability and validity (Cronbach's alpha = 0.94). The 14 items on the BSQ-14 scale represent the feelings of BID and body-shape anxiety. Three cognitive variables made up the questionnaire were self-cognition, dietary impairment, and behavioral change. Since every item is worded negatively, the responders suffer more BID the higher the BSQ score. For example, the first item, “Have you ever worried about your size and think you should diet?” assigns up to six points: “never” = 1, “little” = 2, “sometimes” = 3, “often” = 4, “very often” = 5, and “always” = 6. The total score of 14 items is from 14 to 84, and the higher the total score shows the more body image dissatisfaction.

### Statistical analysis

IBM SPSS Statistics (version 23.0) was used for statistical analysis. Differences in quantitative data between sexes were compared using two independent samples Student's *t test*. One-way analysis of variance (ANOVA) was used to analyze differences in quantitative variables in different puberty stages. Pearson correlation and multiple linear regression models were adopted to analyze the relations between BID and BAIs. The level of statistical significance was set at *P* < 0.05.

## Results

There were 329 boys and 280 girls included in the study. Of them, 174 students were in stage 1, 206 students were in stage 2, and 229 students were in stage 3. As shown in Table 1, the results of the *t*-test showed that there were no significant differences in age, BSQ scores, height, weight, CC, WC, WHtR, CSHR, HSHR, or BMI between boys and girls (*P* > 0.05). The SH, HC, SST, TST, AST, HHR, and SHHR among boys were significantly lower than those among girls (*P* < 0.05). The WHR and WSHR among boys were significantly higher than those among girls (*P* < 0.05).

**Table 1:** The comparisons of BSQ score, anthropometric indices between boys and girls (*x* ± SD).

	**Boys (*n =* 329, $\bar{x}$ ±SD)**	**Girls (*n =* 280, $\bar{x}$ ±SD)**	***t***	***p***
Age	10.85 ± 1.77	11.11 ± 1.80	−1.80	0.07
BSQ	24.40 ± 12.54	26.10 ± 13.01	−1.64	0.10
Height	151.59 ± 12.91	151.46 ± 11.16	0.13	0.90
SH	80.05 ± 6.20	81.26 ± 6.02	−2.44	0.02
Weight	45.61 ± 13.28	45.91 ± 13.42	−0.28	0.78
CC	72.04 ± 9.70	72.20 ± 8.52	−0.21	0.84
WC	67.11 ± 10.92	65.71 ± 9.08	1.71	0.09
HC	79.43 ± 9.60	81.06 ± 9.61	−2.08	0.04
SST	15.94 ± 6.73	20.11 ± 7.24	−7.36	<0.01
TST	14.10 ± 7.56	17.41 ± 7.65	−5.36	<0.01
AST	17.19 ± 9.31	20.29 ± 9.29	−4.09	<0.01
WHtR	0.44 ± 0.06	0.43 ± 0.05	1.95	0.05
HHR	0.52 ± 0.04	0.53 ± 0.04	−3.01	<0.01
WHR	0.84 ± 0.07	0.81 ± 0.07	5.91	<0.01
CSHR	0.90 ± 0.09	0.89 ± 0.07	1.72	0.06
WSHR	0.84 ± 0.12	0.81 ± 0.10	3.38	<0.01
SHHR	0.53 ± 0.01	0.54 ± 0.01	−7.85	<0.01
HSHR	0.99 ± 0.08	0.99 ± 0.08	−0.73	0.47
BMI	19.51 ± 3.63	19.87 ± 5.20	−0.99	0.32

**Figure 1:**
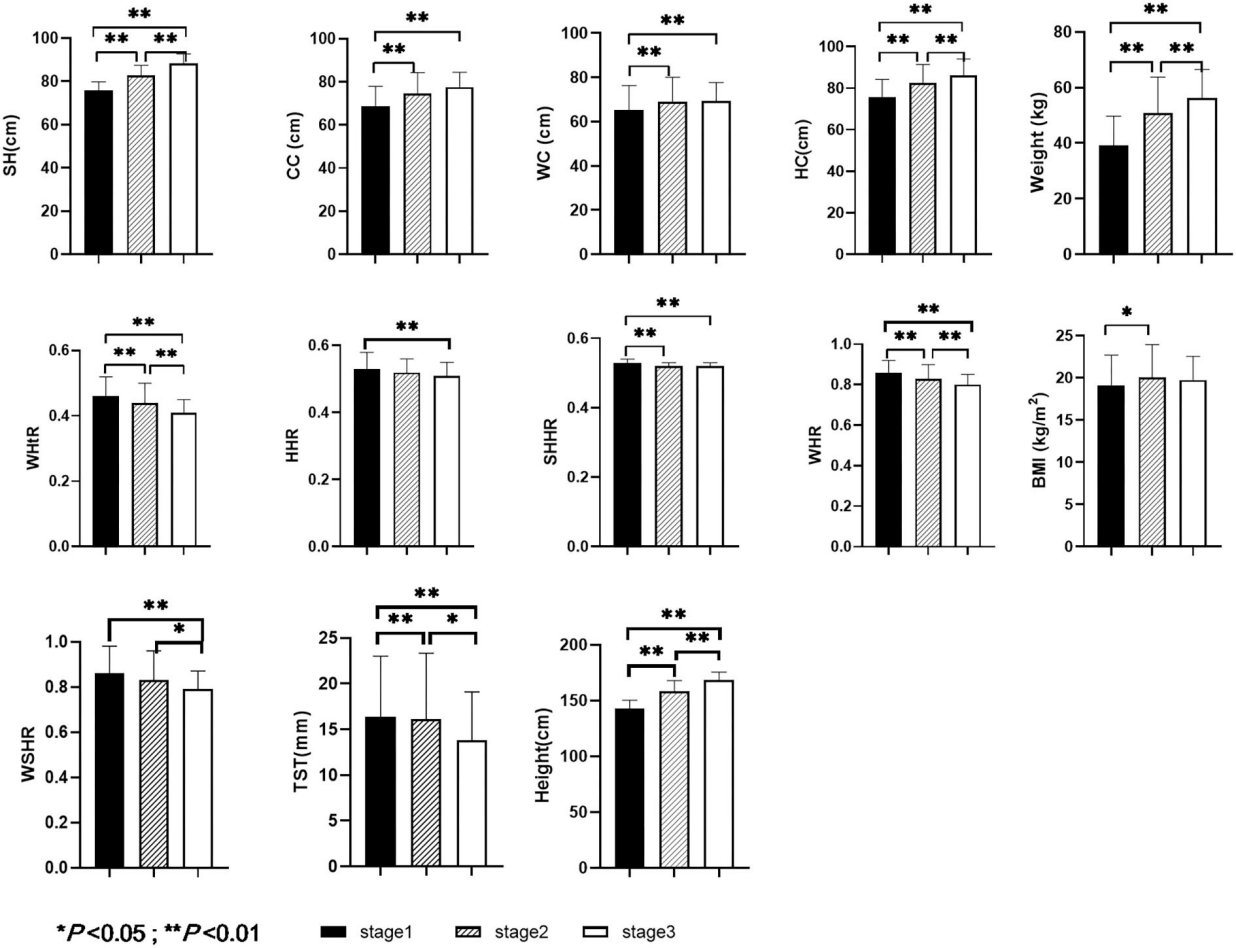
Comparisons of height, SH, CC, WC, HC, WHtR, HHR, SHHR, WHR, BMI, WSHR, TST, and weight among boys with different pubertal development stages.

As shown in Figure 1, the ANOVA results showed that there were no significant differences in BSQ score, SST, AST, CSHR, or HSHR among boys in different development stages (*P* > 0.05). There were significant differences in height, SH, CC, WC, HC, WHtR, SHHR, WHR, BMI, weight, TST, HHR, and WSHR among boys in different development stages (*P* < 0.05).

**Figure 2:**
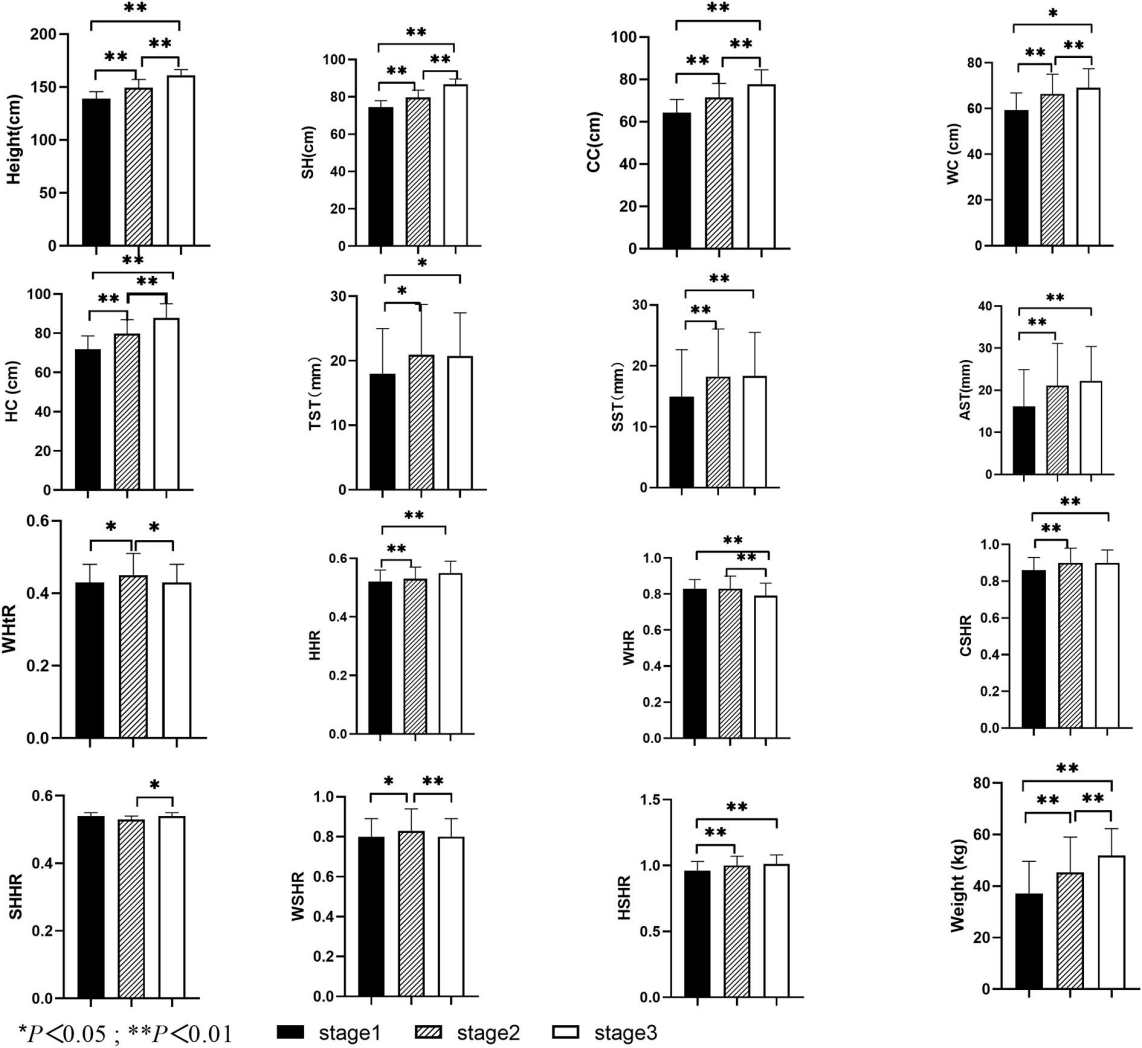
Comparisons of height, SH, weight, CC, WC, HC, TST, AST, SST, HSHR, WHtR, HHR, WHR, CSHR, WSHR, SHHR among girls with different pubertal development stages.

As shown in Figure 2, there were no significant differences in BSQ score or BMI among girls in different development stages (*P* > 0.05). There was a significant difference in height, SH, weight, CC, WC, HC, TST, AST, SST, HSHR, WHtR, HHR, WHR, CSHR, WSHR, and SHHR among girls in different development stages (*P* < 0.05).

As shown in Table 2, Pearson correlation was adopted to analyze the relations between BID and BAIs. As shown in Table 3, the BAIs significantly associated with the BSQ score were used as independent variables and the BSQ score was used as a dependent variable. Multiple linear regression models were conducted.

**Table 2:** Pearson correlation coefficients of associations between body anthropometric indices and BSQ score.

**Variables**	**Boys**			**Girls**		
	**Stage 1 (*n =* 166)**	**Stage 2 (*n =* 119)**	**Stage 3 (*n =* 44)**	**Stage 1 (*n =* 75)**	**Stage 2 (*n =* 87)**	**Stage 3 (*n =* 118)**
Height	0.114	0.114	0.236	0.110	0.238^*^	−0.01
SH	0.225^**^	0.124	0.290	0.119	0.206	0.143
Weight	0.486^**^	0.561^**^	0.641^**^	0.161	0.370^**^	0.440^**^
CC	0.563^**^	0.588^**^	0.568^**^	0.288^**^	0.606^**^	0.473^**^
WC	0.573^*^	0.597^**^	0.516^**^	0.380^**^	0.504^**^	0.498^**^
HC	0.529^**^	0.574^**^	0.546^**^	0.302^**^	0.546^**^	0.450^**^
SST	0.447^**^	0.603^**^	0.482^**^	0.364^**^	0.582^**^	0.310^**^
TST	0.484^**^	0.690^**^	0.499^**^	0.341^**^	0.533^**^	0.375^**^
AST	0.475^**^	0.594^**^	0.396^**^	0.360^**^	0.567^**^	0.338^**^
WHtR	0.593^**^	0.619^**^	0.482^**^	0.377^**^	0.475^**^	0.526^**^
HHR	0.589^**^	0.655^**^	0.506^**^	0.330^**^	0.554^**^	0.491^**^
WHR	0.430^**^	0.404^**^	0.256	0.307^**^	0.222^*^	0.254^**^
CSHR	0.558^**^	0.628^**^	0.453^**^	0.286^*^	0.601^**^	0.457^**^
WSHR	0.565^**^	0.618^**^	0.456^**^	0.373^**^	0.478^**^	0.483^**^
SHHR	0.231^**^	0.013	0.168	0.018	−0.055	0.223^*^
HSHR	0.546^**^	0.653^**^	0.489^**^	0.328^**^	0.577^**^	0.449^**^
BMI	0.541^**^	0.666^**^	0.620^**^	0.131	0.310^**^	0.450^**^

* P < 0.05;

** P < 0.01.

**Table 3:** The results of multiple linear regressions on associations between body anthropometric indices and body image dissatisfaction.

**variables**	**β**	***SE***	***t***	***P***
**Boys with stage 1**				
HHR	8.17	0.88	9.25	<0.01
**Boys with stage 2**				
HHR	2.51	1.23	2.05	0.04
SST	4.98	1.24	4.00	<0.01
**Boys with stage 3**				
Weight	10.30	1.27	4.99	<0.01
**Girls with stage 1**				
WHtR	5.12	1.39	3.69	<0.01
**Girls with stage 2**				
CSHR	10.82	1.64	6.57	<0.01
WHR	−3.61	1.74	29.54	0.04
**Girls with stage 3**				
WHtR	6.09	0.92	6.60	<0.01
SHHR	2.05	0.89	2.30	0.02

### Boys

In stage 1, the results of the Pearson correlation showed that body weight, CC, WC, HC, SST, TST, AST, WHtR, HHR, CSHR, WSHR, HSHR, SH, SHHR, WHR and BMI was significantly positively correlated with BSQ score (*P* < 0.05). The results of multiple linear regression showed that the HHR was significantly correlated with BSQ score (*P* < 0.01).

In stage 2, the results of the Pearson correlation showed that body weight, CC, WC, HC, SST, TST, AST, WHtR, HHR, CSHR, WSHR, HSHR, WHR and BMI was significantly positively correlated with BSQ score (*P* < 0.05). The results of multiple linear regression showed that the HHR and SST were significantly correlated with BSQ score (*P* < 0.05).

In stage 3, the results of the Pearson correlation showed that body weight, CC, WC, HC, SST, TST, AST, WHtR, HHR, CSHR, WSHR, HSHR and BMI was significantly positively correlated with BSQ score (*P* < 0.05). The results of multiple linear regression showed that weight was significantly correlated with BSQ score (*P* < 0.01).

#### Girls

In stage 1, the results of the Pearson correlation showed that the CC, WC, HC, SST, TST, AST, WHtR, HHR, WHR, CSHR, WSHR, and HSHR were significantly positively correlated with BSQ scores (*P* < 0.05). The results of multiple linear regression showed that the WHtR was significantly correlated with BSQ score (*P* < 0.01).

In stage 2, the results of the Pearson correlation showed that the CC, WC, HC, SST, TST, AST, WHtR, HHR, WHR, CSHR, WSHR, height, weight, BMI and HSHR were significantly positively correlated with BSQ scores (*P* < 0.05). The results of multiple linear regression showed that CSHR and WHR were significantly correlated with BSQ score (*P* < 0.05).

In stage 3, the results of the Pearson correlation showed that the CC, WC, HC, SST, TST, AST, WHtR, HHR, WHR, CSHR, WSHR, weight, BMI, SHHR and HSHR were significantly positively correlated with BSQ scores (*P* < 0.05). The results of multiple linear regression showed that WHtR and SHHR were significantly positively correlated with BSQ score (*P* < 0.05).

## Discussion

Adolescence is the key stage of body image cognition and body shape development (25, 26). Since children's body shape development characteristics are different at different development stages, their body shape concerns may also be different. The results of the present study showed that the anthropometric indicators (in boys with stage 1: HHR; in boys with stage 2: HHR, SST; in boys with stage 3: weight; in girls with stage 1: WHtR; in girls with stage 2: CSHR, WHR; in girls with stage 3: WHtR, SHHR) associated with BID were different among Chinese children and adolescents at different developmental stages.

This study showed positive associations between HHR and BSQ scores in boys with testicular volume < 4 ml (stage 1), which means boys with larger hip circumferences were more dissatisfied with their body image. At this stage, boys' puberty has not yet started, and HHR may reflect adolescents' perception of their body shape better than the fat percentage. Muscle volumes do not increase quickly in normal children (24). At a young age, children not only grow up but also expand their hip circumference, and it is a normal growing process. At the same time, many studies found that hip circumference is associated with the visceral fat area in children. This means that hip circumference can predict childhood normal body growth and adiposity (27). Most Chinese parents think their children's fat is a sign of health and affluence, especially among boys (28), and since parents may provide high-calorie and high-fat foods to make their children grow large, the growth of HHR in children with stage 1 may not be the increase of muscle but may be more reflective of the accumulation of upper body adipose tissue. It was reported that 8-year-old boys believe that muscle quality, strength, and speed were related to the ideal male image (29). Boys hope to be leaner, stronger, and more muscular (30). In boys who have not started puberty, the positive association between HHR and BID may be due to the accumulation of more fat in the upper body, which also indicates that boys in stage 1 may pay attention to the development of the upper body. The positive association between HHR and body image dissatisfaction may be due to the accumulation of more fat.

In boys with testicular volume ≥4 ml and non-first spermatorrhea (stage 2), HHR and SST were positively correlated with the BSQ score, which means that boys with a larger HHR and SST were more dissatisfied with their body image. In boys in stage 2, male secondary sexual characteristics have begun to develop. As puberty advances, androgens begin to increase, promoting bone and muscle growth and development, especially resulting in changes in height and hips. At the same time, this stage is accompanied by an increase in upper body fat (31). Studies have shown that the idealized physical criteria for boys at this stage are tall stature, strength, and muscular and wide shoulders (32). A higher HHR reflects a shorter and thicker figure, and a higher SST reflects greater upper body fat accumulation. The results of this study demonstrate that boys at stage 2 may pay more attention to their hips, height, and upper body fat.

In boys after the first spermatorrhea (stage 3), weight was positively associated with BSQ score, which means boys with greater body weight were more dissatisfied with their body image. In boys at stage 3, sexual organs have matured, and their growth begins to slow down. Some studies have shown that boys with mature sexual organs have higher requirements for their bodies and gain V-shaped bodies and extra muscles (32). Boys with mature sexual organs pay more attention to body image and want to change their bodies (bigger or thinner) to meet the standard of beauty and to increase the attractiveness of their bodies. Previous studies have shown that children who are overweight or obese are more dissatisfied with their bodies than those with normal weight (4). Therefore, boys in stage 3 may pay more attention to their weight.

In girls with breast development < Tanner stage II (stage 1), WHtR was positively associated with BSQ score, which means that girls with a higher WHtR were more dissatisfied with their body image. The girls in the first stage of pubertal development have not yet started puberty. The WHtR reflects the degree of abdominal fat accumulation. Previous studies have shown that abdominal fat accumulation is associated with a devaluation of appearance (33). The results of this study were consistent with those of earlier studies.

In girls with breast development ≥ Tanner stage II and non-menarche (stage 2), the CSHR was positively correlated with the BSQ score, but the WHR was negatively associated with the BSQ score, which means that girls with a higher CSHR or a lower WHR were more dissatisfied with their body image. This seems to be inconsistent with the cognitive view of adult women that an “s-shaped” body is beautiful (34). Girls at the stage 2 of pubertal development have started puberty, and their female secondary sexual characteristics have begun to develop. It is well known that estrogen begins to increase after the onset of puberty, promoting breast development and hip fat accumulation (35). To some extent, the CSHR reflects the development of the breasts. The WHR reflects the accumulation of abdominal fat, but it also reflects the development of hips in adolescent girls (36). This may be because girls who have just started puberty are not ready for the rapid development of female characteristics, such as breasts and hips. They are not prepared for the objective and sexual attention caused by the start of puberty. Girls who have just started puberty cannot incorporate their body image from the perspective of mature women (30). They may be more concerned with their maladjustment to the rapid development of their bodies.

In girls after menarche (stage 3), the WHtR and SHHR were positively associated with the BSQ score, which means that girls with a higher WHtR and SHHR were more dissatisfied with their body image. In girls at stage 3 of pubertal development, sexual organs have matured and their growth has begun to slow down. The WHtR is an important index that reflects abdominal obesity, and the SHHR is an index that reflects the ratio of trunk length to lower limbs (36). Most girls with earlier menarche expected to become thinner, despite their weight being at a healthy level. Previous studies have shown that WC is associated with BID (37). In addition, women are also expected to have better body proportions and longer legs, which is consistent with the findings of this study. Therefore, the ideal body image for girls after menarche lies in slimness and better body proportions.

There were several limitations. First, this study is a cross-sectional study. The associations between anthropometric indices and BID at different developmental stages need to be verified by cohort studies. Second, in revealing the association between anthropometric indices and BID, parents, partners, film and television media and other factors related to body image were not adjusted. Third, the medical staff who received standardized training asked whether the child had experienced the first spermatorrhea or menarche, which may be biased.

## Data availability statement

The raw data supporting the conclusions of this article will be made available by the authors, without undue reservation.

## Ethics statement

The studies involving human participants were reviewed and approved by the Human Research Ethics Committee of the Bengbu Medical College (Ethics Approval Number: 2015 No. 003). Written informed consent to participate in this study was provided by the participants' legal guardian/next of kin.

## Author contributions

YW and RC: conceptualization, date curation, formal analysis, writing-original draft. XP: date curation and methodology. LZ and ZZ: investigation and methodology. LF: data curation, writing—original draft, writing—review and editing, and project administration. All authors contributed to the article and approved the submitted version.
